# Delivery of Gemcitabine Prodrugs Employing Mesoporous Silica Nanoparticles

**DOI:** 10.3390/molecules21040522

**Published:** 2016-04-21

**Authors:** Alessio Malfanti, Ivana Miletto, Emanuela Bottinelli, Daniele Zonari, Giulia Blandino, Gloria Berlier, Silvia Arpicco

**Affiliations:** 1 Dipartimento di Scienza e Tecnologia del Farmaco, Università di Torino, Via P. Giuria 9, 10125 Torino, Italy; alessio.malfanti89@gmail.com (A.M.); daniele.zonari@unito.it (D.Z.); giulia.blandino.gb@gmail.com (G.B.); 2 Dipartimento di Chimica and NIS (Nanostructured Interfaces and Surfaces) Centre, Università di Torino, Via P. Giuria 7, 10125 Torino, Italy; ivana.miletto@uniupo.it (I.M.); embottinelli@hotmail.com (E.B.)

**Keywords:** mesoporous silica nanoparticles, drug delivery systems, anticancer drugs, prodrugs, biomaterials, surface chemistry

## Abstract

In this paper, mesoporous silica nanoparticles (MSNs) were studied as vehicles for the delivery of the antitumoral drug gemcitabine (GEM) and of its 4-(*N*)-acyl derivatives, (4-(*N*)-valeroyl-(C5GEM), 4-(*N*)-lauroyl-(C12GEM) and 4-(*N*)-stearoyl-gemcitabine (C18GEM)). The loading of the GEM lipophilic prodrugs on MSNs was explored with the aim to obtain both a physical and a chemical protection of GEM from rapid plasmatic metabolization. For this purpose, MSNs as such or with grafted aminopropyl and carboxyethyl groups were prepared and characterized. Then, their different drug loading capacity in relation to the nature of the functional group was evaluated. In our experimental conditions, GEM was not loaded in any MSNs, while C12GEM was the most efficiently encapsulated and employed for further evaluation. The results showed that loading capacity increased with the presence of functional groups on the nanoparticles; similarly, the presence of functional groups on MSNs’ surface influenced the drug release profile. Finally, the cytotoxicity of the different preparations was evaluated and data showed that C12GEM loaded MSNs are less cytotoxic than the free drug with an activity that increased with the incubating time, indicating that all these systems are able to release the drug in a controlled manner. Altogether, the results demonstrate that these MSNs could be an interesting system for the delivery of anticancer drugs.

## 1. Introduction

Cancer is currently the second leading cause of death in the world after cardiovascular diseases [1], while it probably shows the highest clinical complexity. Chemotherapy remains the mainstay of cancer therapy. However, the employed anticancer agents still present some drawbacks, limiting their efficacy: most cytotoxic drugs have low specificity, because they do not discriminate between cancerous and normal cells, leading to the onset of systemic toxicity and to adverse effects that limit treatment efficacy [2].

The emerging discipline of nanomedicine, that brings nanotechnology and medicine together in order to develop novel therapies and improve existing treatments, could provide an effective answer to the complexity of the cancer disease [3]. Nanomedicine offers additional therapeutic options compared to present conventional therapy and a large number of nanocarriers possessing the ability to carry and deliver therapeutic or diagnostic agents to the disease site are under development for applications related to cancer diagnosis and treatment [4,5,6,7]. Different anticancer drug delivery systems (such as liposomes, albumin bound nanoparticles, polymeric nanoparticles, polymeric micelles, and cyclodextrins) have moved into the clinic or are in clinical trials, while others are still under study [8].

In recent decades, the development of new techniques and synthetic strategies led to the discovery of new bioactive molecules and researchers have tried to extend their knowledge on developing new drug delivery nanocarriers. Among these vehicles, “nanoinorganic” systems have been studied both for therapeutic and diagnostic purposes [9,10,11,12]. In recent decades, mesoporous silica nanoparticles (MSNs) have been the subject of intense research [13,14,15,16,17,18,19]. Compared to traditional organic nanocarriers (such as liposomes or other colloidal systems), these vehicles exhibit unique properties of inorganic nanomaterials, such as thermal and chemical stability and ease of chemical modification of surface silanol groups [20]. MSNs show very interesting properties for application in the development of drug delivery devices, such as stable mesoporous structure, high specific surface area (600–1000 m^2^/g), large pore volume (0.6–1 cm^3^/g), regular and tunable mesopore diameters (1.6−30 nm) and a pore channel system homogeneously organized in hexagonal mesostructures. These features are the basis of their potential as nanocarriers, thanks to the huge available volume where molecules can be encapsulated and released. Moreover, the fabrication of MSNs is simple, scalable and controllable. The biocompatibility of these materials at different levels, including cells, tissues and animals, was demonstrated by several groups. Many types of MSNs have been shown to be nontoxic in many biological systems if they are prepared with certain optimized structural features and are applied in the right dosage [15,21,22,23]. Moreover, it has been reported that MSNs exhibit lower hemolytic activity compared to their nonporous counterparts [24,25], this property having great significance for intravenous administration of these drug nanocarriers. In general, the exhaustive evaluation of compatibility and safety of MSNs requires deep investigations on the physicochemical properties, such as particle size and morphology, pore size and arrangement and surface properties that can all play important roles in their interactions with biological systems [26,27,28,29].

Gemcitabine (2′,2′-difluoro-2′-deoxycytidine, GEM) is an anticancer nucleoside analog of deoxycytidine that is active against solid tumors, including colon, lung, pancreatic, breast, bladder, and ovarian cancers [30,31,32]. Moreover, GEM is the first-line chemotherapeutic agent since 1996 against advanced or metastatic pancreatic cancer [33]. GEM is a pyrimidine antimetabolite with a chemical structure similar to that of cytarabine, except that two geminal fluorine atoms are placed in the 2′ position of the deoxyribose sugar ring. Once inside the cell, GEM needs to be phosphorylated by deoxycytidine kinase into its active form, the 5′-triphosphategemcitabine, which is incorporated into the DNA strand, halting its elongation and causing cell death. Moreover, GEM action also involves ribonucleotide reductase inhibition [34,35]. At the same time, GEM is rapidly metabolized in the blood, liver and kidneys as a consequence of deamination by cytidine deaminase into the chemotherapeutically-inactive uracil derivative [36,37,38]. Thus, when administered intravenously, GEM has a very short plasma half-life, which represents a major limitation of this anticancer compound [39,40,41]. Many different approaches have been evaluated to improve GEM metabolic stability and its *in vitro* and *in vivo* cytotoxic activity. To provide physical stability, GEM was encapsulated in liposomes and in nanoparticles [42]. Alternatively, the synthesis of various GEM derivatives was attempted in order to chemically protect the 4-amino group of the drug from the metabolic inactivation [43].

In the last few years, some multifunctional MSNs for GEM delivery have been proposed [44,45,46]. The controlled and tumor-specific drug release was achieved by coating the nanoparticles with different compounds such as: lipids, serum albumin or pH sensitive polymers [47,48,49].

The aim of this work is to explore the ability of different MSNs to be employed as a convenient vehicle for the delivery of antitumor drugs. For this purpose, we present the synthesis and functionalization of MSNs and the further encapsulation of GEM and of its lipophilic prodrugs. The technological properties of these formulations are discussed, with a preliminary evaluation of their *in vitro* antitumor activity.

## 2. Results and Discussion

### 2.1. Synthesis and Characterization of Mesoporous Silica Nanoparticles (MSNs)

**Figure 1:**
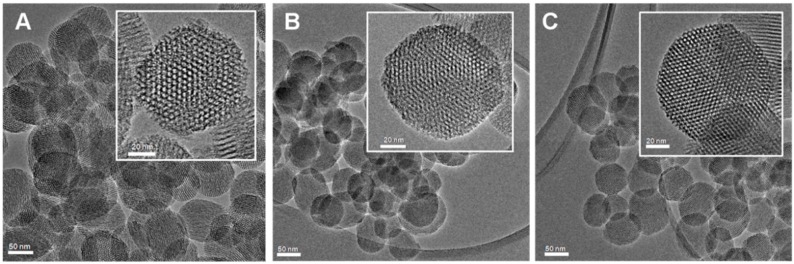
HRTEM images of (**A**) MSN; (**B**) Carboxy-MSN; (**C**) Amino-MSN.

MSNs were prepared by a sol-gel procedure in the presence of the surfactant cetyltrimethylammonium bromide (CTAB) as structure directing agent and functionalized by post-synthesis grafting as previously reported [50,51]. The obtained materials exhibited quasi-spherical particle morphology with an average particle size of *ca.* 100 ± 23 nm and regular and ordered cylindrical channels with hexagonal symmetry. Representative High Resolution Transmission Electron Microscopy (HRTEM) images of the samples are reported in Figure 1.

According to the results from gas-volumetric analyses (N_2_ adsorption/desorption isotherms at liquid nitrogen temperature), as expected, MSNs showed a type IV isotherm, typical of mesoporous materials with one-dimensional cylindrical channels [52,53] and a Specific Surface Area (SSA) of *ca.* 1200 m^2^/g with average pore diameter of *ca*. 3.7 nm, in the case of the as-synthesized material. A decrease in SSAs, average pore diameters and volumes was detected in the case of the Amino and —in lesser amount—for Carboxy-functionalized materials, as reported in Table 1. The maintenance of the regular mesoporous structure after functionalization was assessed also by HRTEM and XRD analysis, whilst the presence of the functional groups on the surface of Amino-MSN and Carboxy-MSN was confirmed by thermogravimetric analysis (TGA) and FTIR measurements (Table 1 and Materials and Methods for details). These data suggest that the functional groups are dispersed inside the pores and on the external surface of the particles, thus influencing their interactions with drug molecules and physiological media. Indeed, a recent work from some of us showed how the materials show different surface charge and hydrophilic character [54]. Moreover, when compared to other silica-based materials, bare MSN showed a relatively low water uptake, which was interpreted by assuming a not-homogeneous surface Si-OH distribution, resulting in the presence of both hydrophobic and hydrophilic regions on the material surface (see [54] and references therein).

### 2.2. Drug Loading

The drug loading efficiencies of the different MSNs were determined by HPLC. We observed that GEM was not loaded in any MSNs, whatever the tested ratios and incubation time.

In order to evaluate the dependence of the loading efficiency on the solvent, the reaction was also conducted by incubating MSNs in acetone or methanol and adding GEM dissolved in a small amount of water. In these conditions, GEM was also not loaded in any MSNs, suggesting that the competition with the solvent (water has a relatively high affinity for the silica surface) is not the main parameter affecting the encapsulation process.

On the contrary, the physico-chemical features of the drug could influence its encapsulation in silica nanoparticles. For example, the GEM log *p*-value is lower than that of drugs that have been successfully encapsulated into MSNs such as paclitaxel or doxorubicin [55,56]. In fact, the GEM log *p*-value is −1.4 while paclitaxel and doxorubicin log *p*-value is 3 and 1.27, respectively. This is, in some way, surprising, since silica is expected to be hydrophilic. However, the same trend regards the molar volume which was calculated to be around 142 cm^3^ for GEM, 610 cm^3^ for paclitaxel and 336 cm^3^ for doxorubicin. This parameter could affect loading efficiency—for instance, it has been reported that a suitable ratio between pore and molecular size could improve the loading process [22].

**Figure 2:**
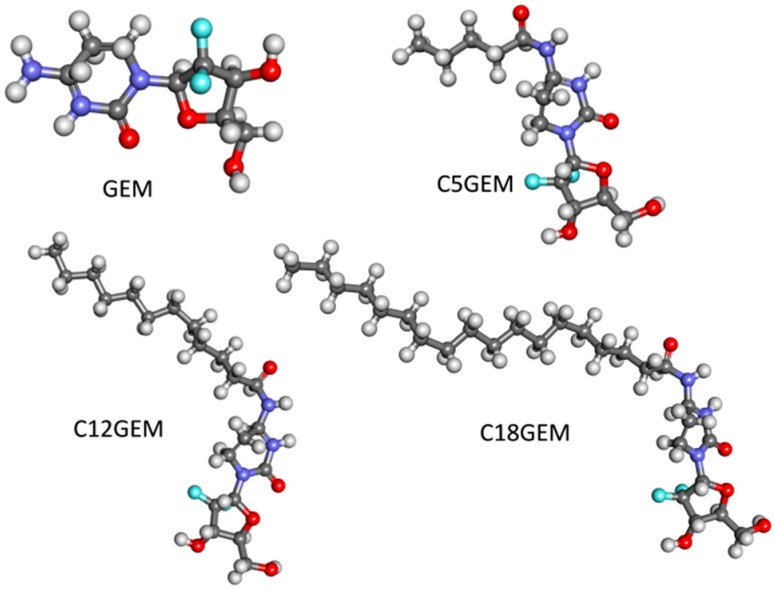
Ball and stick representation of gemcitabine and its prodrugs: carbon atoms are in grey, oxygen in red, nitrogen in violet, fluorine in light blue, hydrogen in light grey. Visualization and fast geometry optimization were obtained with the Discovery Studio Visualizer software (v.16.1.0.15350, San Diego, CA, USA of Biovia), by employing a Dreiding-like forcefield.

In order to increase the steric hindrance of GEM, we have previously synthesized a series of its lipophilic prodrugs: 4-(*N*)-valeroyl-(C5GEM), 4-(*N*)-lauroyl-(C12GEM) and 4-(*N*)-stearoyl-gemcitabine (C18GEM) (Figure 2). These lipophilic derivatives showed a higher encapsulation efficiency in liposomes and polymeric nanoparticles in comparison to the parent drug and were characterized by a good *in vitro* and *in vivo* activity [57,58,59,60]. Moreover, the calculated log *p*-value of the prodrugs is higher than that of the parent drug (1.10 for C5GEM, 4.74 for C12GEM and 7.91 for C18GEM) [60]; thus, we decided to evaluate the interaction between these prodrugs and the MSNs with different functional groups.

The drug loading experiments were done at different time intervals and various GEM prodrugs:MSNs *w*/*w* ratios were evaluated. The loading efficiency of C5GEM and C18GEM was lower than that obtained with the encapsulation of C12GEM, indicating that a proper interplay between the drug lipophilic character and size influence the loading process. This is indeed a complex phenomenon ruled by many factors such as diffusion, competition with the solvent, steric hindrance and weak (dispersion, van der Walls, hydrogen bonding, hydrophobic) interactions.

Moreover, C12GEM showed the highest *in vitro* cytotoxic activity among the three prodrugs tested, and, thus, further experiments were performed using this derivative.

The results related to the more efficient C12GEM:MSNs ratio (2:1 *w*/*w*) at different time intervals using the various MSNs are shown in Table 2. The data show that the drug loading efficiency increases in the case of functionalized MSNs, in the order Carboxy- > Amino- > MSNs. Some considerations can be done by comparing the size of the drug with that of the three materials pores. GEM is a small molecule, with the longer dimension around 11 Å, which is similar to Ibuprofen, often employed as a model drug for encapsulation in mesoporous silica based materials. The size of C12GEM could be estimated to be around 20 × 11 Å, without considering a folding of the C12 chain, which would make these values smaller. This size is thus perfectly compatible with the accessible pore size of the three materials, which is on the order Amino- (27 Å) < Carboxy- (31 Å) < MSN (37 Å). Moreover, it is clear that surface functionalization improved the loading capacity, so that specific hydrophobic and hydrogen bonding interactions with functional groups (and related alkyl chains) are playing an important role in the interaction with the lipophilic prodrugs hosted in the MSN pores.

It was also observed that the drug loading efficiency is dependent on the incubation time—in particular, when increasing the time of incubation, the drug loading efficiency decreases. A similar phenomenon was observed by Vallet-Regi *et al.* [61] and was explained on the basis of molecular hindrance of the drug and the weakness of the bonds between drug and MSNs. The adsorption process is ruled by weak surface interactions and by the diffusion of the drug and solvent molecules to and from the inner surface of silica. We can thus infer that the resulting host/guest complex is not thermodynamically stable. This means that prolonged stirring could provide the energy for drug release from the material pores.

### 2.3. In Vitro Drug Release Study

**Figure 3:**
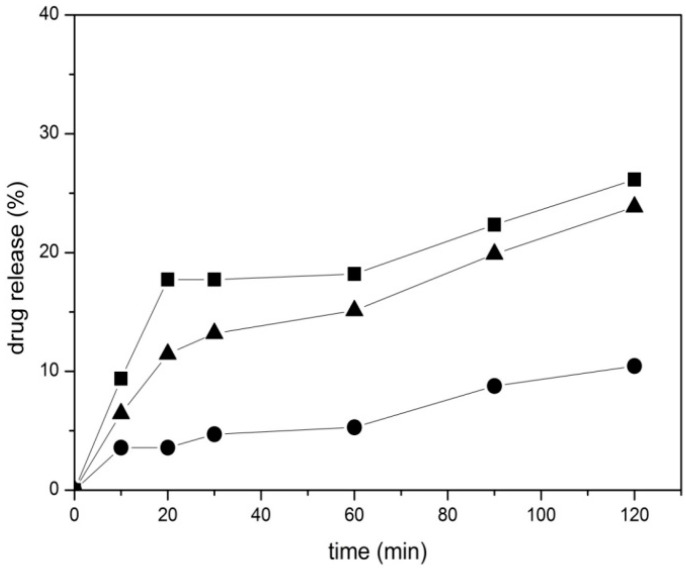
*In vitro* release profiles of MSN-C12GEM ■ Amino-MSN-C12GEM ▲ and Carboxy-MSN-C12GEM ● in PBS medium at 37 °C. All experiments were done in triplicate. SD: ±10%.

*In vitro* C12GEM release experiments from drug loaded MSNs were carried out by soaking samples in PBS buffer at 37 °C. The release values shown in Figure 3 were calculated as percentage with respect to the drug loading measured in the three complexes (Table 2). All samples show a very gradual and slow release, which is influenced by the presence of surface functional groups. In particular, the release rate of the MSNs was the fastest one, followed by Amino-MSNs and by Carboxy-MSNs as the lowest. This indicates that the diffusion of the prodrug from the three samples to the buffer solution depends on the functionalization with a trend inverse to that found in the drug loading experiments. Carboxy-MSNs showed the highest drug loading (23 μg/mg) and the lowest release in percentage, suggesting a relatively higher interaction between C12GEM and the functional groups. On the contrary, MSNs were able to encapsulate the lowest drug amount and released them quickly, with the Amino-functionalized sample showing and intermediate behavior.

The weak interactions taking place with surface silanol, amino or carboxylate groups are thus playing an important role in the drug loading and release processes. However, we underline the fact that the amount of C12GEM released by the three samples after 120 min is similar, being around 1.85 μg for MSNs and Carboxy-MSNs and 2.5 μg for Amino-MSNs.

### 2.4. Cytotoxicity

We evaluated the cytotoxicity of GEM and C12GEM on a panel of different cancer cell lines, and we decided to use two cell lines for our experiments that showed a different sensitivity towards these compounds. Therefore, the *in vitro* cytotoxic activity was tested on MDA-MB-231 (human breast adenocarcinoma) and A2780 (human ovarian carcinoma) cells at different time (24, 48 and 72 h).

**Figure 4:**
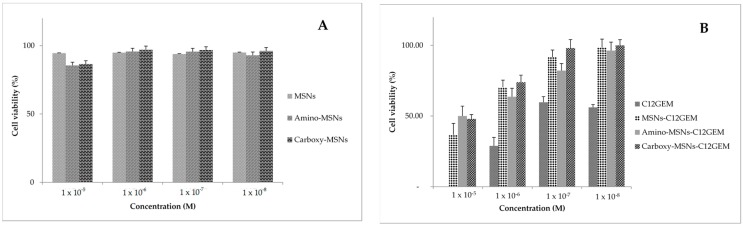
Cytotoxicity of (**A**) unloaded MSNs; and (**B**) C12 GEM loaded MSNs against MDA-MB-231 cells after 72 h of incubation.

**Figure 5:**
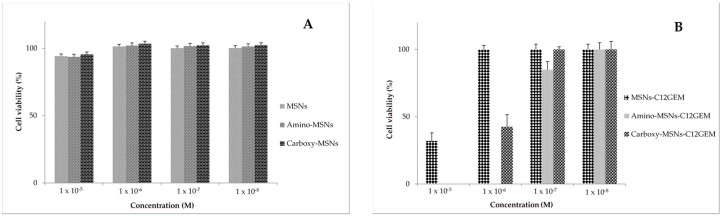
Cytotoxicity of unloaded (**A**) MSNs; and (**B**) C12 GEM loaded MSNs against A2780 cells after 72 h of incubation.

The cytotoxicity of unloaded MSNs was previously evaluated and MSNs, Amino-MSNs and Carboxy-MSNs showed almost no toxicity on MDA-MB-231 and A2780 cells at the considered concentration range, indicating that all nanoparticle types are biocompatible nanocarriers (Figure 4A and Figure 5A).

The cytotoxicity of GEM and of its lipophilic prodrug was also evaluated. The results reported in Table 3 show that C12GEM was more toxic than GEM on A2780 cells whilst the toxicity of the two molecules is quite similar on MDA-MB-231. This behavior is similar to that obtained with other cell lines [57,60], indicating that acylation of amino group of GEM, by reducing the catabolic effect of cytidine deaminase, enhanced the prodrug’s cytotoxic activity in comparison with the parent drug. From these data, it is also possible to observe that GEM and C12GEM are more active on A2780 in comparison with MDA-MB-231 cells.

Finally, the cytotoxic activity of C12GEM loaded MSNs was evaluated (Table 3, Figure 4B and Figure 5B). The data show that prodrug cytotoxicity was reduced after encapsulation in MSNs. However, the cytotoxic activity of the complex increases with the increasing of incubation time, indicating that the MSNs effectively release and gradually deliver the cytotoxic compound into the cancer cells. This is important since the encapsulation of C12GEM could be exploited to further increase the stability of the prodrug.

## 3. Materials and Methods

### 3.1. Materials

Carboxyethylsilanetriol sodium salt (25% in water) was purchased from abcr GmbH & Co. (Karlsruhe, Germany) and used as received. Gemcitabine was purchased from TCI Europe (Zwijndrecht, Belgium). All the other reagents and solvents were purchased from Sigma-Aldrich (Milan, Italy) and used as received. The 4-(*N*)-acyl derivatives of gemcitabine were synthesized according to Immordino *et al.* [57].

### 3.2. Synthesis and Functionalization of MSN

MSN sample was prepared by using CTAB as the structure directing agent (SDA) and tetraethyl orthosilicate (TEOS) as the silica source according to a slightly modified literature procedure [62,63]. CTAB (1 g, 2.74 mmol), was dissolved in 480 mL of deionized water under stirring and heating. At the stable temperature of 80 °C, NaOH (2.0 M, 3.5 mL) was slowly added to the mixture. TEOS (5 mL, 22.4 mmol) was then added dropwise over 10 min under vigorous stirring. After 2 h of stirring at 80 °C the milky reaction mixture was cooled to room temperature (RT) and the white precipitate was filtered off and washed with abundant water and methanol. The SDA was removed from the as-synthesized material by calcination at 550 °C, heating to the desired temperature under N_2_ flow and switching to O_2_ for a 6 h isotherm.

Amino- and Carboxy-MSN samples were prepared by post-synthesis grafting employing 3-aminopropyl triethoxysilane (APTS) and carboxyethylsilanetriol, respectively, as described in the following. 1 g of calcined MSN (overnight dried at 100 °C) were suspended in 30 mL of anhydrous toluene. The particles suspension was heated at 130 °C under stirring. Next, 0.6 mL (0.5676 g, 2.56 mmol) of 3-aminopropyl triethoxysilane (APTS) were added drop-wise and the mixture was allowed to reflux for 17 h. The reaction solution was then cooled down, filtered and washed with toluene. The obtained white solid was cured in air at 120 °C overnight.

Carboxy-MSNs were prepared by suspending 1 g of calcined MSNs in 50 mL of methanol. 2 mL of carboxyethylsilanetriol (25% in water, 1.19 mmol) were added under vigorous stirring and the reaction mixture was stirred at RT for 4 h. The reaction mixture was then filtered and washed with methanol. The obtained white solid was cured in air at 120 °C overnight.

### 3.3. Characterization of MSNs

HRTEM analyses were performed by means of a JEM 3010-UHR microscope (JEOL Ltd., Tokyo, Japan) operating at 300 kV. For the measurements, MSN powders were dispersed on a copper grid coated with a perforated carbon film. The size distribution of the samples was obtained by measuring a statistically representative number of particles (*ca.* 200 particles) and the results are indicated as mean particle diameter (dm) ± standard deviation (STD) (dm ± STD).

**Figure 6:**
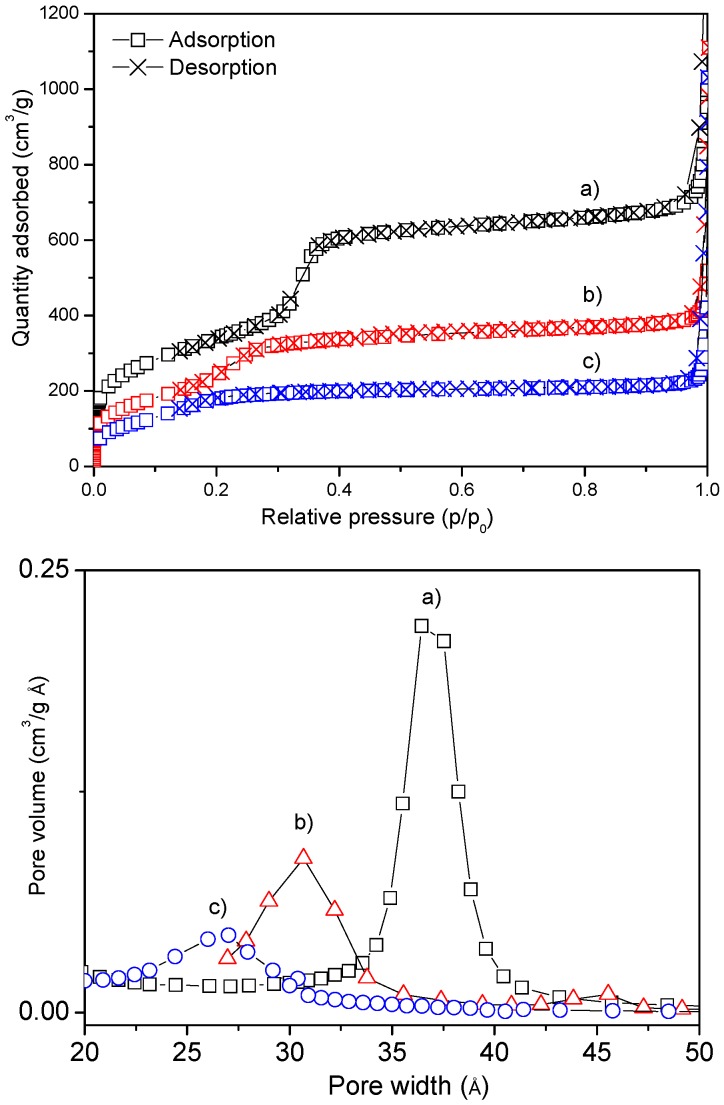
(**Top**) nitrogen adsorption and desorption isotherms on (**a**) MSN; (**b**) Carboxy-MSN; and (**c**) Amino-MSN; (**Bottom**) corresponding pore size distributions.

SSA was measured by N_2_ adsorption-desorption isotherms at 77 K using a Micromeritics ASAP2020 instrument (Micromeritics Italy SRL, Milano, Italy). The SSA was calculated by the Brunauer–Emmet–Teller (BET) method and the average pore size according to the Barrett–Joyner–Helenda (BJH) method, employing the Kruk–Jaroniec–Sarayi equations on the adsorption branch of the isotherms. Before the analyses, the samples were outgassed at RT overnight. Curves in top panel of Figure 6 (top) can be classified as type IV isotherms, due to the presence of a steep adsorption increase below p/p^0^ = 0.4, which is characteristic of capillary condensation inside mesopores. This feature shifts to lower p/p^0^ values after functionalization with carboxy and amino groups, which corresponds to a decrease in the pore size. This in fact corresponds to a change in the pore size distributions in the three materials (Figure 6, bottom panel).

**Figure 7:**
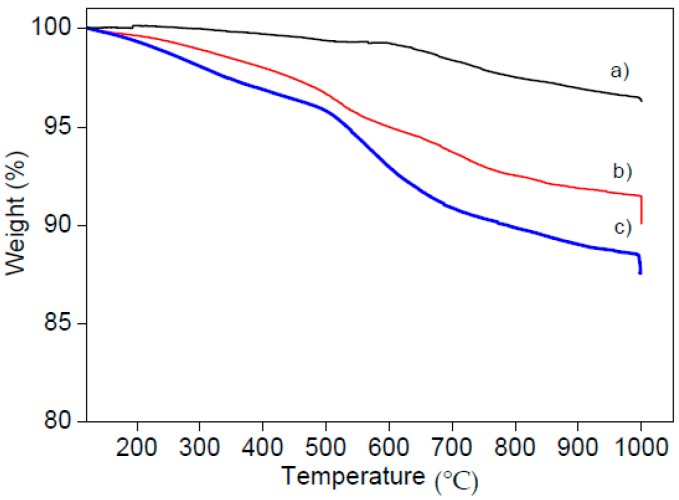
TGA profiles of (**a**) MSN; (**b**) Carboxy-MSN; and (**c**) Amino-MSN.

Thermal gravimetric analysis (TGA) was carried out on a TAQ600 (TA Instruments, Milano, Italy) by heating the samples, after equilibration, from 30 to 1000 °C at a rate of 10 °C/min. Once the target temperature was reached, an isotherm was run for 15 min in air in order to burn carbonaceous residues from pyrolysis reactions. TGA curves show three important regions of weight loss from 150 °C upwards, after desorption of physically adsorbed water (Figure 7). The first one up 400/500 °C and the third from 600/700 °C to 1000 °C is due to dehydroxylation. The decomposition of the organic modifiers is measured in correspondence of the second weight loss from ~420 °C to ~600 °C for carboxy-MSN and from ~500 °C to ~700 °C for amino-MSNs, by adding the vertical loss at 1000 °C related to combustion of hydrocarbons generated during pyrolysis in nitrogen atmosphere.

**Figure 8:**
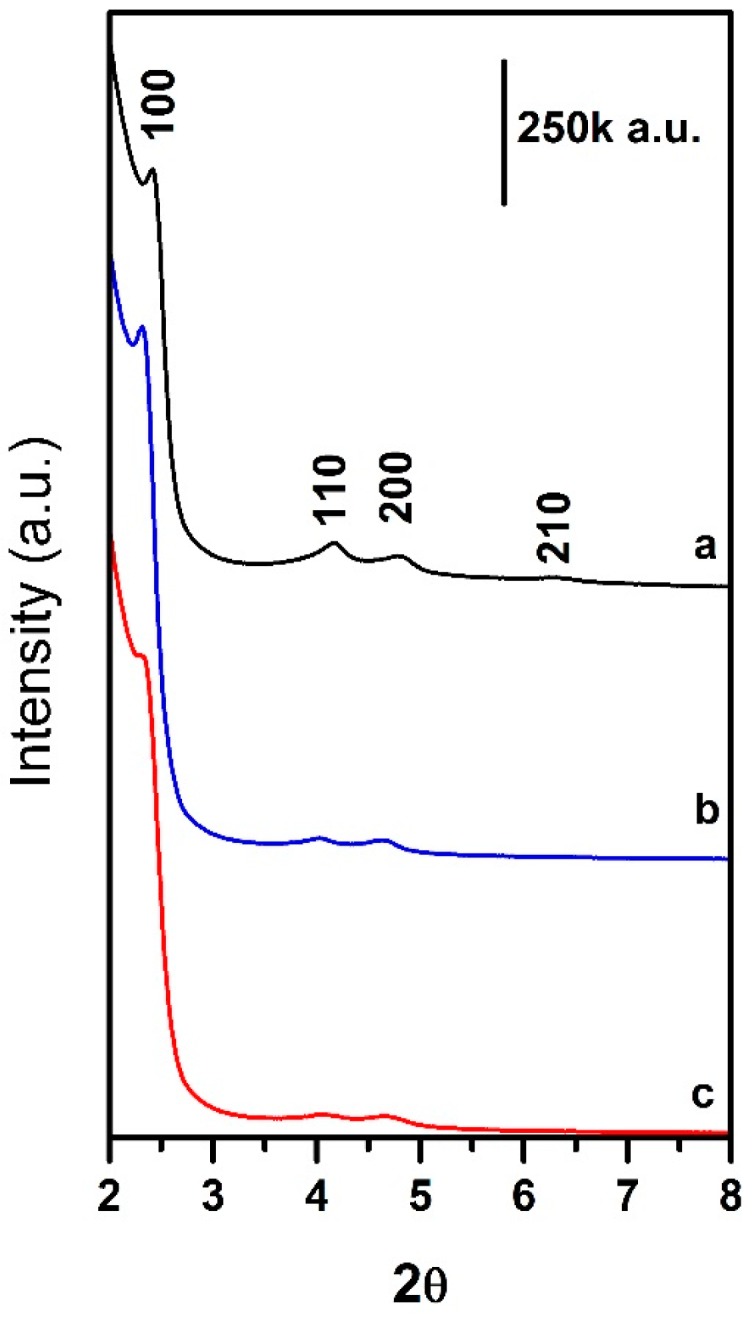
Powder XRD patterns of MSNs (**curve a, black line**), Carboxy-MSNs (**curve b, blue line**) and Amino-MSNs (**curve c, red line**).

Powder X-ray diffraction (XRD) patterns of MSNs were collected on a PANalyticalX’Pert PRO (PANalytical Srl, Milano, Italy) instrument operating with Cu Ka radiation (1.54 Å), generated at 45 kV and 40 mA. MSN sample shows the typical (100), (100), (200) and (210) peaks related to the hexagonal array of pores, typical of MCM-41 mesoporous materials (Figure 8). After functionalization, the peaks’ intensity decrease noticeably, and their position is modified, as often observed after functionalization. This suggests the lining of the functional groups within the pore walls.

**Figure 9:**
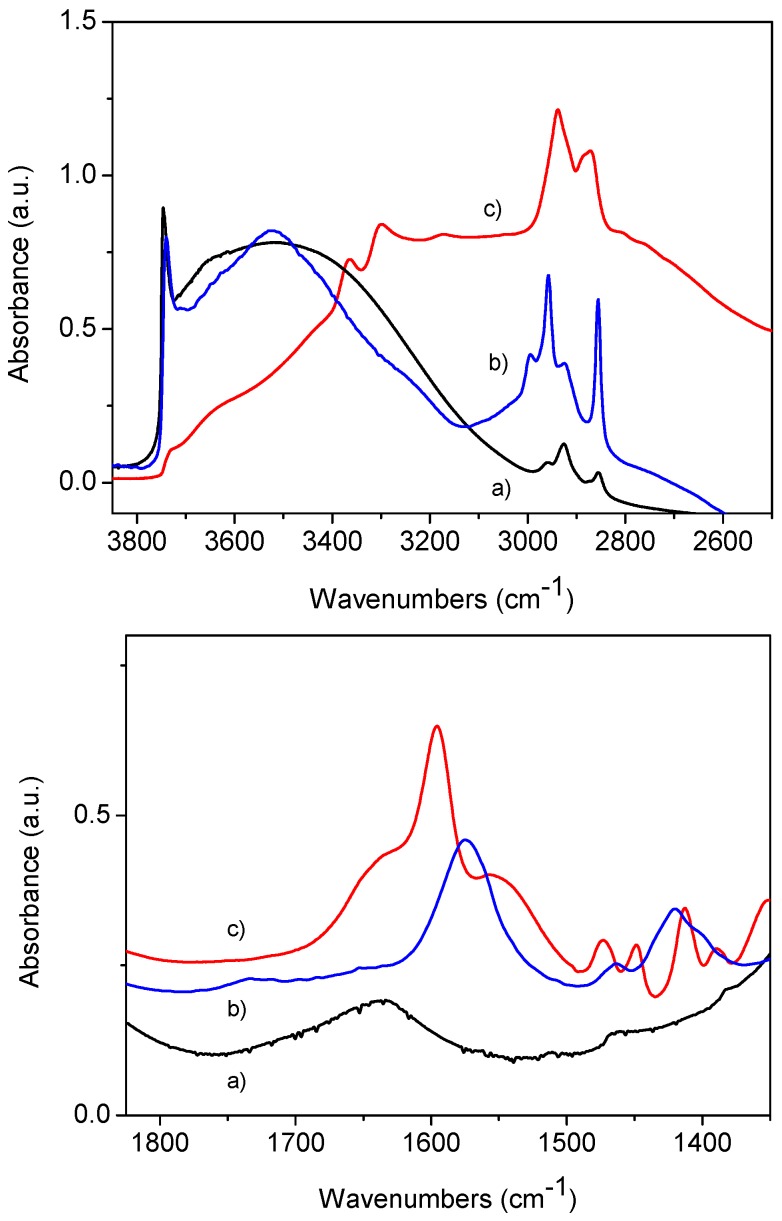
FTIR spectra in the high and low frequency region (top and bottom, respectively) of: (**a**) MSN; (**b**) Carboxy-MSN; and (**c**) Amino-MSN.

Fourier Transform infrared spectra (FTIR) were recorded on a Jasco-5300 spectrometer (Jasco Europe Srl, Cremella, LC, Italy) equipped with a DTGS detector, working with 4 cm^-1^ resolution over 32 scans. Samples were in form of self-supporting pellets suitable for transmission IR experiments and placed in a quartz cell equipped with KBr windows and designed for RT studies in vacuum and in controlled atmosphere. Before analysis the samples were outgassed at RT to remove physically adsorbed water. In the high frequency range (top panel of Figure 9) it is possible to appreciate the decrease of the band 3745 cm^−1^ related to the stretching vibration of isolated surface SiOH (νOH) after functionalization. This is almost quantitative for Amino-MSN where the bands related to νNH_2_ and νCH_2_ groups can be seen between 3400 and 3200 and below 3000 cm^−1^, respectively. The corresponding bending modes (δNH_2_ and δCH_2_) are seen at 1595 and below 1500 cm^−1^, respectively (Figure 9, bottom panel). In the Carboxy-MSN sample the band related to silanols is relatively intense (top panel), but the success of functionalization is clear from the intense νCH_2_ modes below 3000 cm^−1^, and by the presence of two bands at 1572 and 1414 cm^−1^, related to the antisymmetric and symmetric stretching modes of COO^−^ groups, respectively (bottom).

### 3.4. Gemcitabine (GEM) and GEM Prodrugs Loading

GEM (3 mg, 0.01 mmol) was mixed with the different MSNs in different ratios (1:1, 1:2, 2:1 *w*/*w*) into 1200 µL of distilled water. A stock solution of GEM in water was also prepared and incubated with MSNs diluted in acetone or methanol. The solution was stirred and samples of 400 µL each were taken at different time intervals (2, 5 and 24 h). Then, the mixtures were centrifuged, the supernatant was removed and GEM loaded MSNs were washed with 200 µL of distilled water.

The same procedure was used for the loading of the different gemcitabine prodrugs, but acetone (for C5GEM and C12GEM) or isopropyl alcohol (for C18GEM) were used instead of distilled water, due to different solubility of the molecules.

In order to determine the amount of GEM into MSNs, 1 mg of the drug-loaded samples were resuspended in distilled water and sonicated for 40 min, to favor the extraction of the encapsulated drug. Then, the samples were centrifuged at 10,000 rpm for 3 min and the supernatants were measured by HPLC. To evaluate the amount of GEM prodrugs in MSNs, the samples were diluted in acetone or isopropyl alcohol and ultrasonicated. After centrifugation, the supernatants were evaporated under nitrogen flow and the white powder obtained was dissolved in methanol and quantified by HPLC.

To confirm the drug loading efficiency, after the loading reaction, the supernatant and the washing solutions were collected and the residual GEM or GEM prodrugs amounts were measured by HPLC. The amount of drug loaded was calculated based on its initial and residual concentration.

The HPLC system consisted of a Shimadzu LC-10ADvp pump and a Shimadzu SPD-10Avp UV–Vis detector (Shimadzu, Kyoto, Japan); the analytical column was a LiChroCART C18 (250 mm × 4 mm i.d., 5 µm particle size) equipped with a C18 column guard (Merck, Milan, Italy). For GEM, the column was eluted with ammonium acetate/methanol (95:5 *v*/*v*), flow rate 1mL/min, and the elution profile was monitored at 272 nm. The mobile phase was methanol/water (90:10 *v*/*v*) for C5GEM and C12GEM, (flow rate 0.8 mL/min) and methanol for C18GEM (flow rate 1 mL/min), and the elution profile was monitored at 248 nm. Peak heights were recorded and processed on an Autochro Data Module (Young Lin Instrument Co., Anyang, Korea) equipped with the software Autochro-3000 Chromatograph Data System (Young Lin Instrument Co.). The drug concentration was calculated from standard curves. The assay was linear over the tested concentration range (20–1000 ng).

### 3.5. In Vitro Drug Release

The release of the loaded MSNs was performed in 0.1 M phosphate buffer solutions (PBS, pH 7.4) and all the release studies were carried out in triplicate. The loaded MSNs were added to 1200 µL of PBS at 37 °C under magnetic stirring. At a predetermined time intervals, aliquots (100 µL) were taken from the suspension and centrifuged at 14,000 rpm for 3 min. The supernatants were then analyzed by HPLC in the conditions described above. The removed fluid was immediately replaced with an equal amount of fresh medium at the same temperature to maintain sink condition.

### 3.6. Tumor Cell Lines and Cell Culture

The cell lines used were MDA-MB-231 (human breast adenocarcinoma) and A2780 (human ovarian carcinoma). MDA-MB-231 cells were grown in DMEM (Dulbecco’s Modified Eagle Medium) supplemented with 10% fetal calf serum, 0.03% of L-glutamine and 2% penicillin and streptomycin. A2780 cells were maintained in RPMI (Roswell Park Memorial Institute medium) 1640 containing 10% fetal calf serum, 0.03% of L-glutamine, 2% penicillin and streptomycin, and 50 μg/mL of gentamicin sulfate. Cells were maintained in a humidified incubator at 37 °C in 5% CO_2_.

### 3.7. Cell Proliferation Assays

Cell growth inhibition was evaluated by sulforhodamine B colorimetric proliferation assay (SRB) modified by Vichai and Kirtikara [64].

MDA-MB-231 and A2780 cells, maintained as described above, were seeded at 3 × 10^4^ cells/well in 96 wells microtiter plates and incubated overnight to allow cellular adhesion. Various dilutions of GEM, C12GEM, loaded MSNs (expressed as drug concentration) and unloaded MSNs (expressed as MSNs concentration) were added in triplicate, and incubated for 24, 48 and 72 h.

## 4. Conclusions

In this work, we have demonstrated a dependence of the loading ability of bare and functionalized mesoporous silica nanoparticles, MSNs, on molecular properties of the drug, in particular the steric hindrance. The drug molecules could be complexed into the pores of MSNs through van der Waals, hydrophobic forces and hydrogen bonds depending on size and lipophilic character. Our study revealed that, for an efficient complexation in MSNs of GEM prodrugs, the molecule must be mildly lipophilic and sterically hindered. In fact, the hydrophilic drug GEM was not complexed in the different MSNs prepared, irrespective of the employed solvent. On the other hand, the GEM lipophilic prodrug with intermediate size and log *p*-values among the tested ones (C12GEM) was encapsulated in the MSNs at various rates depending on their functional groups and pores dimension.

This approach is very promising, since we are persuaded that the encapsulation of a GEM prodrug in nanoparticles confers a dual protection of the molecule from degradation and rapid elimination.

In this study, it was observed that the MSNs complexed with C12GEM had extended release *in vitro*, and this factor is important for an efficient drug delivery system.

Finally, the toxicity of MSNs was investigated. From the comparison of IC_50_ values, it could be concluded that the encapsulated drug has a weak cytotoxic effect when compared to free drugs on both A2780 and MDA-MB-231 cell lines in the first 24 h. In the following hours, an increase in the nanosystems efficacy was observed.

## Figures and Tables

**Table 1:** General properties of the MSN materials employed for drug complexation.

Sample	Pore Diameter ^1^ (Å)	SSA ^2^ (m^2^·g^−1^)	Porevolume ^1^ (cm^3^·g^−1^)	Functional Groups ^3^ wt %
MSN	37	1239	1.10	-
Amino-MSN	27	704	0.54	6.0
Carboxy-MSN	31	960	0.42	4.3

^1^ Calculated by the BJH method in the adsorption branch, employing the Kruk–Jaroniec–Sayari model; ^2^ Calculated by the BET method; ^3^ Calculated from TGA as described in Materials and Methods (caption of Figure 7).

**Table 2:** Drug loading efficiency of the different MSNs expressed as µg of C12GEM in 1 mg of nanoparticles.

Sample	Incubation Time (h)		
	2	5	24
MSN-C12GEM	7.4	4.8	3.5
Amino-MSN-C12GEM	11	5.7	6.6
Carboxy-MSN-C12GEM	23	16.4	14

**Table 3:** Cytotoxic activity (IC_50_) on human tumor cell lines MDA-MB-231and A2780 at 24, 48 and 72 h ^1^.

Compound			IC_50_ (M)			
		MDA MB231		A2780		
	24 h	48 h	72 h	24 h	48 h	72 h
Gemcitabine	>10^−4^	9.5 × 10^−5^	1.0 × 10^−7^	1.0 × 10^−7^	6.2 × 10^−7^	6.0 × 10^−8^
C12GEM	1.0 × 10^−4^	8.0 × 10^−5^	8.5 × 10^−6^	8.0 × 10^−6^	<10^−9^	<10^−10^
MSN-C12GEM	>10^−5^	>10^−5^	6.0 × 10^−5^	>10^−5^	4.0 × 10^−6^	5.5 × 10^−6^
Amino-MSN-C12GEM	>10^−5^	>10^−5^	1.0 × 10^−5^	8.0 × 10^−5^	7.5 × 10^−6^	8.5 × 10^−6^
Carboxy-MSN-C12GEM	>10^−5^	>10^−5^	2 × 10^−5^	>10^−5^	1.0 × 10^−6^	2.0 × 10^−6^

^1^ The values are arithmetic means of three determinations.

## Abbreviations

The following abbreviations are used in this manuscript:

APTS	3-aminopropyl triethoxysilane
BET	Brunauer–Emmett–Teller
BJH	Barrett–Joyner–Halenda
CTAB	cetyltrimethylammonium bromide
C5GEM	4-(*N*)-valeroyl-gemcitabine
C12GEM	4-(*N*)-lauroyl-gemcitabine
C18GEM	4-(*N*)-stearoyl-gemcitabine
DMEM	Dulbecco’s Modified Eagle Medium
FTIR	Fourier Transform Infrared Spectroscopy
GEM	gemcitabine
HPLC	High Performance Liquid Chromatography
HRTEM	High Resolution Transmission Electron Microscopy
MSN	mesoporous silica nanoparticle
PBS	phosphate buffer saline
RPMI	Roswell Park Memorial Institute Medium
RT	room temperature
SDA	structure direct agent
SSA	specific surface area
STD	standard deviation
TEOS	tetraethyl orthosilicate
TGA	thermogravimetric analysis
XRD	X-ray diffraction
